# Knowledge, attitudes and practices of primary care nurses regarding human immunodeficiency virus pre-exposure prophylaxis in Mhlontlo sub-district municipality, Eastern Cape, South Africa

**DOI:** 10.4102/safp.v67i1.6204

**Published:** 2025-12-18

**Authors:** Olugboyega O. Akingbohungbe, Parimalaranie Yogeswaran, Olukayode A. Adeleke

**Affiliations:** 1 Department of Family Medicine and Rural Health, Faculty of Medicine and Health Sciences, Walter Sisulu University, Mthatha, South Africa

**Keywords:** human immunodeficiency virus, pre-exposure prophylaxis, sexually transmitted infection, primary healthcare, knowledge, attitudes and practice

## Abstract

**Background:**

Pre-exposure prophylaxis (PrEP) is key for preventing human immunodeficiency virus (HIV) transmission, but uptake is limited, especially in rural areas of South Africa. Nurses in primary healthcare (PHC) play a vital role in delivering PrEP, yet little is known about their knowledge, attitudes and practices (KAP) in this area.

**Methods:**

A cross-sectional analytic study was conducted among 178 nurses from three healthcare facilities in the Mhlontlo sub-district of the OR Tambo District, Eastern Cape. A structured, self-administered questionnaire was used to assess demographic variables, PrEP-related knowledge, attitudes and practices.

**Results:**

While 78.7% of participants were aware of PrEP, only 43.3% demonstrated comprehensive knowledge, and just 46.6% had ever initiated a client on PrEP. Community health centre (CHC) nurses demonstrated significantly better knowledge and practice than those in district hospitals (*p* < 0.001). Attitudes were generally positive, with 91.4% agreeing that PrEP is an effective HIV prevention method. However, 73.1% believed it could lead to increased transmission of sexually transmitted infections (STIs). The study identified barriers to PrEP implementation, including lack of training (57.9%), protocol shortages (25.3%) and insufficient time (40.4%).

**Conclusion:**

Primary care nurses demonstrate a high level of awareness and positive attitudes towards PrEP; however, knowledge and practice gaps persist. Primary care providers’ capacity building through targeted training, integration and improved human and healthcare resources is vital to enhancing PrEP implementation for national HIV prevention.

**Contribution:**

This study highlighted the frontline primary care providers’ vital roles in the PrEP for prevention of HIV infection in resource-limited rural healthcare settings.

## Introduction

Human immunodeficiency virus and/or acquired immunodeficiency syndrome (HIV/AIDS) is a pandemic, with an estimated 39.9 million people living with HIV by the end of 2023, particularly in Africa (65%). In 2023 alone, 630 000 people died from HIV-related causes, highlighting its significant spread and resultant chronic health problems.^1^ Many people living with HIV are still having a poor quality of life, which causes ongoing problems and high death rates in many countries. Even with medical progress, new HIV infections remain common. Access to healthcare services is still a big challenge, especially in low-income and rural areas where health services are poor or overcrowded. This makes it difficult for people to get medicines, diagnostic tests and preventive care. Stigma and discrimination also remain major issues that discourage people from seeking help. Moreover, public awareness about HIV prevention is low, and misinformation continues to spread, making it harder to control the disease worldwide.^2,3^

Pre-exposure prophylaxis (PrEP) is a vital medical intervention aimed at preventing HIV infection in individuals at increased risk. Its main goal is to significantly lower the rate of HIV transmission, thereby supporting wider public health efforts to control and eventually eradicate the HIV epidemic.^4^

Since its endorsement by the World Health Organization (WHO) in 2015, PrEP has become a vital part of the global effort to combat HIV. The WHO recommended tenofovir disoproxil fumarate (TDF) based oral PrEP as an additional prevention method for those at high risk of HIV infection, integrating it into a comprehensive prevention strategy. In later years, WHO expanded its guidance to include the Dapivirine vaginal ring for women at high risk in 2021 and long-acting injectable cabotegravir (CAB-LA) for individuals at notable risk in 2022.^5^

The South African National Department of Health launched its PrEP programme in 2016, primarily targeting high-risk populations, which include men who have sex with men (MSM), sex workers, young women and individuals in serodiscordant relationships.^6^ Despite the demonstrated efficacy of oral PrEP for HIV prevention, substantial challenges remain for its successful rollout, particularly among adolescent girls and young women.

Nurses are vital for PrEP implementation in South Africa, serving as the first contact for patients, educating them, conducting HIV tests and counselling. Their training helps assess risks, facilitate access and promote adherence to PrEP.^7^ Primary care nurses, as front liners, must know guidelines, medications, indications, contraindications and side effects and maintain a positive attitude towards HIV-PrEP. The primary healthcare (PHC) providers, especially nurses, who have the first encounter with these patients, play a crucial role throughout the PrEP programme to enhance adherence and ensure optimal outcomes.^8^ This study aimed to assess the knowledge, attitudes and practices of primary care nurses regarding HIV PrEP in the Mhlontlo subdistrict of the OR Tambo District Municipality in South Africa.

### Hypothesis

Good knowledge and positive attitudes of nurses are significantly associated with good practices of HIV-PrEP.

## Research methods and design

### Study design

A cross-sectional analytic study was carried out between May and June 2024. The knowledge, attitudes and practices of primary care nurses regarding HIV PrEP were examined from three pre-selected healthcare facilities.

### Setting

The study was carried out at three healthcare facilities: Dr. Malizo Mpehle Memorial Hospital (DMMMH), Mhlakulo Community Health Centre (MCHC) and Qumbu Community Health Centre (QCHC), all located within the Mhlontlo Health Subdistrict Municipality in South Africa’s OR Tambo District. This predominantly rural subdistrict has a low Human Development Index and limited educational and developmental capacities. The study was conducted in a sub-district where HIV prevention programmes are integrated into PHC services across 25 clinics, two community health centres (CHCs) and three district hospitals. Regular refresher courses and continuous professional education sessions on HIV prevention are organised by the health department. Nurses in the various facilities are periodically sent for these refresher courses, although there are long intervals in-between, and nurses have complained that most of them do not get an opportunity to attend. In the three facilities under study, only Nurse Initiated Management of Antiretroviral Therapy (NIMART)-trained nurses are allowed to prescribe HIV PrEP, although all other categories of nurses are expected to assist in HIV prevention strategies, including counselling and referral.

Dr. Malizo Mpehle Memorial Hospital in Tsolo is the largest of the three district hospitals, offering a broad range of services and serving as a referral centre for two CHCs. The study population includes all nurses at DMMMH, MCHC and QCHC.

### Study population

The study was conducted in three clusters: DMMMH, MCHC and QCHC. These clusters were selected based on convenience. Dr. Malizo Mpehle Memorial Hospital was included because it is the largest of the three district hospitals and serves as a referral centre for the other two hospitals as well as for 25 surrounding clinics and two CHCs. Mhlakulo Community Health Centre and QCHC were chosen because they were the only two available CHCs in the sub-district.

The study population comprised all nurses working at the selected PHC facilities. The total number of nurses was 296; a breakdown is shown in Table 1. Nurses who were on leave during the study period were excluded from the study.

**TABLE 1:** Study population.

Site	Nursing category	Number
DMMMH	Specialist nurse	11
	Professional nurse	11
	Enrolled nurse	48
	Enrolled nurse assistant	52
	**Total**	**186**
MCHC	Specialist nurse	3
	Professional nurse	26
	Enrolled nurse	13
	Enrolled nurse assistant	15
	**Total**	**54**
QCHC	Specialist nurse	2
	Professional nurse	23
	Enrolled nurse	15
	Enrolled nurse assistant	18
	**Total**	**56**

DMMMH, Dr. Malizo Mpehle Memorial Hospital; MCHC, Mhlakulo Community Health Centre; QCHC, Qumbu Community Health Centre.

### Sample size and sampling

The sample size was 178, as demonstrated in Table 2. The sample size for this study was determined using Cochran’s formula, which is appropriate for a single known population. The formula used is shown in Equation 1:


n={(Zα/2)2×P(1−P)}/d2 [Eqn 1]

**TABLE 2:** Sample size.

Site	Population size	Sample size
DMMMH	186	116
Mhlakulo CHC	54	30
Qumbu CHC	56	32

DMMMH, Dr. Malizo Mpehle Memorial Hospital; MCHC, Mhlakulo Community Health Centre; QCHC, Qumbu Community Health Centre.

Sample size (*N*) is calculated using a Z-score of 1.96 for 95% confidence, an expected proportion (P) of 50% because of no prior HIV-PrEP studies on nurses and a margin of error (d) of 5%.

Applying these parameters resulted in an initial sample size of 384 participants.

To account for potential non-responses, 10% was added to the calculated sample size. This adjustment increased the sample size to 422 participants (384 + 38).

### Finite population correction

Given that the total number of nurses in the target population is less than 10 000, a correction formula was applied to refine the sample size. The correction formula is shown in Equation 2:


nf=ni/(1+ni/N) [Eqn 2]

Here, nf represents the modified sample size after correction, ni is the initial unmodified sample size of 422, and *N* denotes the total number of nurses across the three facilities, which is 296.

Using this formula (see Equation 3), the corrected sample size was calculated as follows:


nf=422/(1+422/296)=173.7, rounded to 174 [Eqn 3]

### Allocation of sample size across study sites

The total sample size was allocated proportionally among the three study sites, based on the number of nurses at each location. The probability-proportional-to-size (PPS) sampling method was employed, using the formula in Equation 4:


ni=(n/N)×Ni [Eqn 4]

Here, ni is the sample size for each facility, n is the corrected sample size (174), N is the total nurse population (296), and Ni is the number of nurses at each facility.

This approach ensured that the sample was distributed proportionally, reflecting the number of nurses at each study site.

The sample size calculation incorporated an intraclass correlation coefficient (ICC) to adjust for the potential similarity of individuals within the same facility. A cluster random sampling method was used. The researcher obtained a list of all nurses from the nursing managers of the facilities. Each nurse on the list was assigned a unique number. Using a random number table, participants were then selected from each cluster to ensure fairness and avoid bias. A random number table is a statistical tool that contains a list of random numbers, helping to generate unbiased random data from a specified distribution.

### Data collection

A piloted questionnaire served as the primary data collection tool, designed to evaluate nurses’ knowledge, attitudes and practices concerning HIV PrEP. Because of linguistic diversity, the questionnaire was developed in both English and IsiXhosa. The English version was professionally translated into Isixhosa and then back-translated into English to ensure accuracy and clarity.

The questionnaire comprised four sections: demographic details such as gender, age, marital status, highest professional education qualification and professional category. There were 16 questions on knowledge such as their understanding of PrEP, different types of PrEP available, which of them are being implemented by the health department, which groups of people are recommended to receive PrEP, to itemise the correct HIV drug combination making up PrEP, the route of administration, etcetera. There were 11 questions on attitudes such as how often the participants discuss sexual risk behaviours and sexual orientation of clients with them, do they believe that HIV PrEP is an effective HIV prevention strategy, could PrEP lead to antiretroviral resistance, etcetera. There were 12 questions on practices related to HIV-PrEP, such as have they ever referred clients for PrEP? If so, how many clients were referred or initiated on PrEP in the last 12 months? Do they recognise a need for an HIV PrEP refresher course? If so, would they avail themselves of the opportunity? etcetera.

On the first day, at each facility, research assistants conducted an informational briefing about the study and responded to nurses’ questions. Each nurse was given a participant information sheet to help them make an informed decision. Researchers answered questions immediately. Nurses who agreed received the consent form and questionnaires, which they completed and returned without consulting others.

### Data entry and analysis

To classify the knowledge levels, the Modified Bloom’s cut-off points, a well-known method in education, were used.^9^ The cut-off points categorised knowledge levels as follows: good (above 50%) and poor (below 50%). The questions on attitude had ‘Yes’, ‘No’ and ‘Not sure’ answers. ‘Yes’ was scored as 1, while ‘Not sure’ or ‘No’ was scored as 0. There were 11 questions in this category, and it was found that the average score was 6, based on the 50% cut-off point. Participants scoring 6 or more were considered to have a positive attitude, while those who scored below 6 were regarded as having a negative attitude.

A favourable reaction to the practice questions was awarded one mark, while a negative response received 0. The average of 6 emerged as the benchmark in classifying practices. Participants who scored an average mark or above were rated as having good practice, and those who scored below the average were labelled as having poor practice.

The data analysis employed various statistical tests, including Chi-square, Fisher’s Exact and *t*-test, to compare the proportions of demographic and background characteristics in the different KAP levels. The Chi-square *p*-value was utilised when all cells had counts of 5 or more; otherwise, Fisher’s exact test was used.

The *t*-test was employed for comparisons involving two groups, and reporting took note of the Levene’s test for equality of variance. The practice data were further analysed to explore associations with knowledge and attitude scores, providing a comprehensive perspective on how these factors influenced participants’ behaviours. This association was ascertained using Spearman rho correlation coefficients for the total KAP scores. The level of significance was set at a *p*-value of less than 0.05. All statistical analyses were computed using SPSS version 29.

### Ethical considerations

Ethical approval was granted by the Walter Sisulu University Health Sciences and Medical Research Ethics Committee (Permit number 028/2024). Permission from the Eastern Cape Department of Health (ECDOH) and the Health Research Database was secured through their online portal at www.nhrd.health.gov.za. Furthermore, administrative approval to proceed with the study was received from the OR Tambo District Health Department and the management of the three health facilities.

## Results

There were 178 nurses participating in the current study. As demonstrated in Table 3, the majority were female (83.7%) with a mean age of 43 years (standard deviation s.d. = 9). Most were single (52.3%), followed by those married (38.6%). Regarding education, 44.4% held a diploma or BSc in nursing, while only 7.3% were specialist nurses. Approximately 43.8% had received training on HIV-related care, and only 23.6% had worked in an HIV clinic, with a mean duration of work experience of 7.5 years.

**TABLE 3:** Demographic characteristics of the participants (*N* = 178).

Variables	Frequency	%
**Gender**		
Male	29	16.3
Female	149	83.7
**Marital status**	**176**	-
Single	92	52.3
Married	68	38.6
Divorced	3	1.7
Widowed	13	7.4
**Highest educational qualification**		
Nursing auxiliary (Enrolled Nurse Assistant)	43	24.2
Enrolment as a nurse	43	24.2
Diploma in nursing science and midwifery or BSc Nursing	79	44.4
Specialist nurse	13	7.3
**Training on HIV**		
Yes	78	43.8
No	98	55.1
Not sure	2	1.1
**Ever worked in an HIV clinic**		
Yes	42	23.6
No	136	76.4

HIV, human immunodeficiency virus.

### Knowledge of human immunodeficiency virus pre-exposure prophylaxis

The majority (78.7%) of them were aware of HIV-PrEP, and most (83.7%) of the nurses knew it was available at their facilities, as demonstrated in Table 4. However, only 43.3% of the nurses could accurately explain what it is. Most of the participants were aware of oral PrEP (92.1%), but they had limited knowledge of injectable and ring-based forms. Contraindications were poorly understood; 64% of them were unaware of creatinine clearance thresholds, and 69.7% did not know PrEP is contraindicated in children under 10. Table 4 shows that the knowledge of HIV-PrEP was significantly higher among nurses in CHCs (75.8%) compared to those in district hospitals (49.1%) (*p* = 0.001), and among professional and specialist nurses compared to among enrolled or auxiliary nurses and younger nurses (< 41.4 years).

**TABLE 4:** Association between demographic variables and knowledge level of human immunodeficiency virus pre-exposure prophylaxis.

Variables of interest	Good knowledge		Poor knowledge		Cramer’ V coefficient	Chi-Square X *p*
	*n*	%	*n*	%		
**Facility type**						
District hospital	57.0	49.1	59.0	50.9	0.258	0.001
Community health centre	47.0	75.8	6.0	20.0	-	-
**Gender**						
Male	19.0	65.5	10.0	34.5	0.063	0.522
Female	85.0	57.0	64.0	43.0	-	-
**Highest educational qualification**						
Nursing auxiliary	21.0	48.8	22.0	51.2	0.368	< 0.0001
Enrolment as a nurse	14.0	32.6	29.0	67.4	-	-
Diploma in nursing science and midwifery or BSc Nursing	60.0	75.9	19.0	24.1	-	-
Specialist Nurse	9.0	69.2	4.0	30.8	-	-
**Professional category**						
Enrolled nurse assistant	21.0	48.8	22.0	51.2	0.402	< 0.0001*
Enrolled nurse	14.0	32.6	29.0	67.4	-	-
Professional nurse	60.0	75.9	19.0	24.1	-	-
Specialist nurse	9.0	81.8	2.0	18.2	-	-
Operational manager	0.0	0.0	2.0	100.0	-	-
**Training on HIV**						
Yes	50.0	64.1	28.0	35.9	0.143	0.187
No	52.0	53.1	46.0	46.9	-	-
**Ever worked in an HIV clinic**						
Yes	24.0	57.1	18.0	42.9	0.014	0.989
No	80.0	58.8	56.0	41.2	-	-
**Age (years)**	41.4	± 8.6	44.9	± 9.6	-	0.012†
**Duration of working as a nurse (years)**	10.0	6.0–14.0	9.0	5.0–14.0	-	0.720‡
**Duration of working in the HIV clinic (years)**	8.0	2.0–10.0	4.0	2.0–10.0	-	0.442‡

HIV, human immunodeficiency virus.

† , Student *t*-test

‡ , Mann–Whitney *U*-test.

* Fisher’s Exact

### Attitudes towards human immunodeficiency virus pre-exposure prophylaxis

Most of the nurses (91.4%) believed PrEP is effective, and 89.2% considered it essential in HIV prevention, as demonstrated in Table 5. However, 73.1% raised concerns that it could increase sexually transmitted infection (STI) transmission, and 51.5% believed it might lead to antiretroviral therapy (ARV) resistance. Despite this, 61% disagreed that PrEP encourages risky sexual behaviour.

**TABLE 5:** Attitudinal characteristics of the participants.

Variables	*n*	%
**Frequency of sexual risk behaviour discussions**		
Some of the consultations	33	19.3
Every consultation	133	77.8
Not at all	5	2.9
**Frequency of sexual orientation discussions**		
Always	119	68.0
Sometimes	38	21.7
Not at all	9	5.1
Not sure	9	5.1
**PrEP is an effective measure to prevent HIV infection**		
Yes	159	91.4
No	15	8.6
**PrEP is a necessary HIV prevention strategy**		
Yes	157	89.2
No	19	10.8
**PrEP increases sexual risk behaviour**		
Yes	100	61.0
No	64	39.0
**PrEP could lead to an increase in STIs among patients**		
Yes	128	73.1
No	47	26.9
**PrEP could lead to ARV resistance**		
Yes	86	51.5
No	81	48.5
**A condom alone is sufficient to prevent HIV infection**		
Yes	85	49.4
No	87	50.6
**PrEP use is associated with significant side effects**		
Yes	69	39.9
No	35	20.2
Not sure	69	39.9
**HIV PrEP’s user adherence is important**		
Yes	157	89.2
No	6	3.4
Not sure	13	7.4
**All nurses in PHC facilities should prescribe HIV PrEP**		
Yes	108	61.7
No	48	27.4
Not sure	19	10.9

PHC, primary healthcare; HIV; human immunodeficiency virus; PrEP, Pre-exposure prophylaxis; ARV, antiretroviral (therapy); STI, sexually transmitted infection.

Sexual health discussions were frequent between nurses and patients. 77.8% of the nurses reported initiating such conversations in all consultations. Most (61.7%) believed all PHC nurses should be allowed to prescribe PrEP. Table 6 shows that positive attitude towards HIV-PrEP was significantly (p< 0.005) influenced by the level of education, professional category HIV training.

**TABLE 6:** Association between demographic variables and attitude towards human immunodeficiency virus pre-exposure prophylaxis.

Variables of interest	Positive attitude		Negative attitude		Cramer’s V coefficient	X^2^ *p*-value
	*n*	%	*n*	%		
**Facility type**						
District hospital	58.0	50.0	58.0	50.0	0.123	0.136
Community health centre	39.0	62.9	23.0	37.1	-	-
**Gender**						
Male	15.0	51.7	14.0	48.3	0.025	0.902
Female	82.0	55.0	67.0	45.0	-	-
**Marital status**						
Single	51.0	55.4	41.0	44.6	0.195	0.065*
Married	41.0	60.3	27.0	39.7	-	-
Divorced	1.0	33.3	2.0	66.7	-	-
Widowed	3.0	23.1	10.0	76.9	-	-
**Highest educational qualification**						
Nursing auxiliary	21.0	48.8	22.0	51.2	0.262	0.006*
Enrolment as a nurse	16.0	37.2	27.0	62.8	-	-
Diploma in Nursing Science and Midwifery or BSc Nursing	54.0	68.4	25.0	31.6	-	-
Specialist nurse	6.0	46.2	7.0	53.8	-	-
**Professional category**						
Enrolled nurse assistant	21.0	48.8	22.0	51.2	0.262	0.009*
Enrolled nurse	16.0	37.2	27.0	62.8	-	-
Professional nurse	54.0	68.4	25.0	31.6	-	-
Specialist nurse	5.0	45.5	6.0	54.5	-	-
Operational manager	1.0	50.0	1.0	50.0	-	-
**Training on HIV**						
Yes	51.0	65.4	27.0	34.6	0.225	0.011
No	44.0	44.9	54.0	55.1	-	-
**Ever worked in an HIV clinic**						
Yes	28.0	66.7	14.0	33.3	0.136	0.102
No	69.0	50.7	67.0	49.3	-	-
**Age (years)**	42.3	± 9.9	43.4	± 8.2	-	0.425†
**Duration of working as a nurse (years)**	10.0	6.0–14.0	8.0	5.0–13.0	-	0.071‡
**Duration working in HIV clinic (years)**	8.0	1.0–10.0	5.0	2.0–10.0	-	0.699‡

† , Welch’s *t*-test

‡ , Mann–Whitney *U*-test

* Fisher’s Exact.

### Practice of human immunodeficiency virus pre-exposure prophylaxis

Fewer than half of the nurses (46.6%) had initiated clients on PrEP, and many of them had done so for fewer than 25 clients. Referral and follow-up rates were low; over half had never referred a client or monitored them on PrEP. Similarly, counselling on side effects and required tests was limited. Practice was better among nurses at CHCs (69.4%) compared to district hospitals (33.6%). It was also better among those with prior HIV training (56.4%) compared to those without (36.7%) as shown in Table 7.

**TABLE 7:** Association between demographic variables and practice level of human immunodeficiency virus pre-exposure prophylaxis.

Variables of interest	Good practice		Poor practice		Cramer’s V coefficient	X^2^ *p value*
	*n*	%	*n*	%		
**Facility type**						
District hospital	39.0	33.6	77.0	66.4	0.342	< 0.0001
Community health centre	43.0	69.4	19.0	30.6	-	-
**Gender**						
Male	13.0	44.8	16.0	55.2	0.011	1.000
Female	69.0	46.3	80.0	53.7	-	-
**Highest educational qualification**						
Nursing auxiliary	20.0	46.5	23.0	53.5	0.098	0.643*
Enrolment as a nurse	17.0	39.5	26.0	60.5	-	-
Diploma in Nursing Science and Midwifery or BSc Nursing	40.0	50.6	39.0	49.4	-	-
Specialist Nurse	5.0	38.5	8.0	61.5	-	-
**Professional category**						
Enrolled nurse assistant	20.0	46.5	23.0	53.5	0.102	0.781*
Enrolled nurse	17.0	39.5	26.0	60.5	-	-
Professional nurse	40.0	50.6	39.0	49.4	-	-
Specialist nurse	4.0	36.4	7.0	63.6	-	-
Operational manager	1.0	50.0	1.0	50.0	-	-
**Training on HIV**						
Yes	44.0	56.4	34.0	43.6	0.227	0.014
No	36.0	36.7	62.0	63.3	-	-
**Ever worked in an HIV clinic**						
Yes	22.0	52.4	20.0	47.6	0.070	0.446
No	60.0	44.1	76.0	55.9	-	-
**Age (years)**	42.9	± 9.2	42.7	± 9.2	-	0.909*
**Duration of working as a nurse (years)**	9.5	7.0–14.0	9.0	5.0–14.0	-	0.584‡
**Duration working in HIV clinic (years)**	8.0	4.0–10.0	3.0	1.0–10.0	-	0.399‡

HIV; human immunodeficiency virus.

† , Fisher’s Exact

‡ , Mann–Whitney *U*-test

* Student *t*-test

Table 6 shows that positive attitude towards HIV-PrEP was significantly (*p* < 0.005) influenced by the level of education, professional category and HIV training.

**FIGURE 1:**
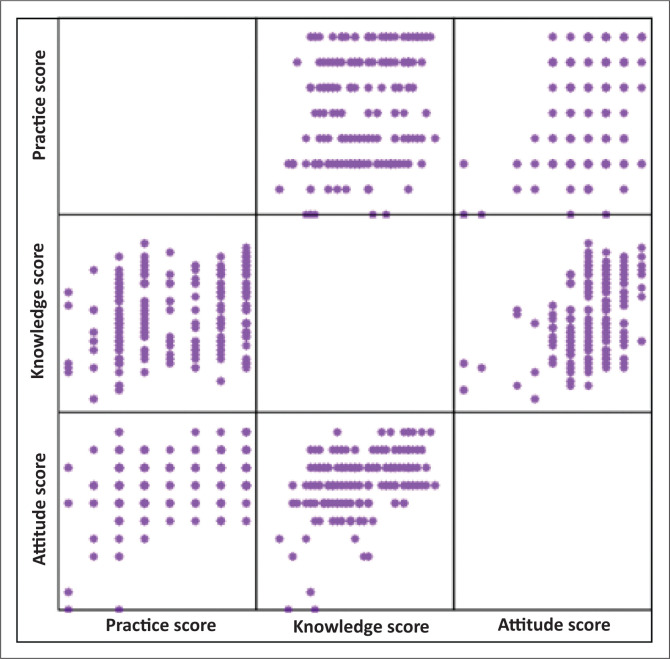
Correlation matrix between the practice, knowledge and attitude of HIV-pre-exposure prophylaxis among 178 primary healthcare providers.

Table 8 presents the correlation between total scores for KAP. The relationships were significant at an alpha level of 0.01, indicating a strong correlation between the KAP. The Spearman’s rho coefficient for practice, knowledge and attitude scores was less than 0.3, indicating that practice was weakly influenced by the health professional’s knowledge and attitude. However, knowledge was moderately influenced (Spearman’s rho coefficient ≥ 0.3) by attitude towards HIV-PrEP. Fig 1 represents the correlation matrix of the KAP of HIV PrEP among the primary healthcare providers.

**TABLE 8:** Correlation between the practice, knowledge and attitude of HIV-pre-exposure prophylaxis among 178 primary healthcare providers.

Variables	Spearman’s Rho	Practice score	Knowledge score	Attitude score
Practice score	Correlation Coefficient	1.000	-	-
	Sig. (2-tailed)	-	-	-
Knowledge score	Correlation Coefficient	0.242	1.000	-
	Sig. (2-tailed)	0.001	-	-
Attitude score	Correlation Coefficient	0.246	0.381	1.000
	Sig. (2-tailed)	0.001	< 0.0001	-

Note: Sig. (2-tailed) - value represents the 2-taled *p*-value of the test.

## Discussion

The study findings reflect a primary healthcare workforce that is generally inexperienced with limited exposure to HIV-specific training. Further education and professional development in HIV-PrEP services would be beneficial. The results also suggest a potential gap in clinical competency that could affect the quality of healthcare services delivered to patients at risk of acquiring HIV. Community Health Centres achieved a 69.4% PrEP initiation rate, significantly outperforming district hospitals, which had a 33.6% rate.

This indicates that CHCs offer a promising model for expanding prevention services. Younger nurses (under 41-years-old) demonstrated better knowledge (*p* = 0.012), suggesting progress in integrating HIV management training into the new cohort of trainees. This improvement is likely because of recent training programmes, updated curricula and the greater engagement of younger professionals with current HIV research. Overall, these findings underline the need for targeted training and interventions to enhance HIV-related knowledge and practice among nurses in primary care.

It was observed that 78.7% of nurses claimed awareness of HIV-PrEP, but only 43.3% could define and explain what it was, thus revealing a knowledge gap. Knowledge varied by age group, educational qualification and professional category. Nurses holding a bachelor’s degree or diploma in nursing, as well as those in the professional category, were better informed (75.9% and 75.8%, respectively). The trends of this study, which suggest that higher scores on PrEP knowledge are linked to professional training and practical experience in HIV care, are supported by previous literature.^10^ Contrary to earlier studies indicating that nurses in HIV-specialised clinics had better PrEP knowledge, this study focused on nurses in general practice settings,^11^ and the findings show that many nurses remain unaware of HIV PrEP contraindications, underlining the need for enhanced training in PrEP provision.^12,13^

Regarding attitude, the study found that nurses with more education about HIV had a more positive attitude. These findings are similar to previous ones that reported that nurses with higher levels of contact and longer experience in nursing HIV patients have more positive attitudes towards PrEP.^14^

According to Karris, Beekmann,^11^ even with the availability of PrEP, prescribing rates among healthcare providers remained low. This was mainly because of inadequate training or a lack of confidence in managing PrEP protocols, similar to what was highlighted. Many healthcare providers complain of a lack of time, unavailable protocols and a shortage of equipment when attempting to implement PrEP services in their practices.

In this study, only 55.2% of the nurses reported ever initiating clients on HIV-PrEP, although many had theoretical knowledge of the intervention. The practice outcome linked to HIV-PrEP training was notably high, as more nurses with such training initiated and monitored clients for PrEP. These findings align with previous research, which emphasises training and direct experience with HIV-PrEP procedures as vital for improving practice outcomes.^15^ However, while they generally seem to be initiating more, the low levels of practice among general healthcare nurses highlight the need for further comprehensive PrEP-specific training for all healthcare providers.

The low PrEP initiation rates are consistent with rural South African studies,^16^ where structural barriers such as stockouts (18%) and protocol shortages (25.3%) hindered implementation. These findings align with Mahlare et al. (15) in identifying systemic resource gaps as critical impediments. However, trained nurses in this study were almost twice as likely to initiate PrEP, supporting MacDonald et al.’s^17^ findings in Uganda, where competency-based training improved delivery by 62%.

Perceived community awareness of PrEP (53.4%) masked significant misinformation, with 22.5% reporting myths like ‘PrEP causes HIV’. Similar gaps were observed in Cape Town, South Africa, where stigma reduced PrEP acceptability by 40%,^18^ and among South African adolescents.^19^ These findings support McLeroy et al.’s^20^ social ecological model, advocating for interventions that link provider training and community education.

This study highlights the need for targeted training programmes for primary care nurses. These programmes should aim at bridging knowledge gaps and equipping nurses with practical skills and confidence to prescribe and manage PrEP effectively. Training should cover not only the clinical aspects of PrEP (such as contraindications, dosage and patient monitoring) but also address common concerns about its use, such as the possibility of promoting risky sexual behaviour, drug interactions and drug resistance.

The limited comprehensive knowledge of PrEP among nurses (43.3% understanding its definition) supports findings in rural sub-Saharan Africa. For example, Eakle et al.^10^ reported that only 48% of South African healthcare providers in primary care could accurately define PrEP, while Mekonnen et al.^12^ observed similar gaps in Ethiopia, where nurses were unaware of renal safety thresholds – a trend reflected in this study (64% were unaware of creatinine clearance criteria). These results highlight systemic deficiencies in standardised training programmes for rural providers.^13^

However, knowledge disparities are greater compared to high-resource settings: only 43.3% of nurses in the Mhlontlo subdistrict demonstrated proficiency, compared with 72% of providers in the United States’ HIV speciality clinics.^11^ This gap highlights inequities in clinical exposure and resource allocation, as specialised clinics often prioritise PrEP training.^21^ Notably, CHC nurses in this study performed better than district hospital peers (75.8% vs. 49.1%), echoing Mack et al.’s^22^ findings in Kenya, where decentralised care encouraged familiarity with prevention tools.

Attitudinal concerns, such as 61% of nurses fearing risk compensation, align with debates in Malawi, where 58% of providers worry about behavioural disinhibition.^23^ There are also gender-based differences in attitudes, with male nurses having a higher likelihood (1.92 times) of positive attitudes. These findings echo Bailey and Moses’ study, which attributed this to socio-cultural norms that limit female nurses’ comfort in discussing sexual health. These concerns starkly contrast with Bavinton and Grulich’s^24^ evidence from Australia and the United States of America. Their PrEP use did not lead to an increase in STI incidence, suggesting that stigma, rather than empirical data, influences perceptions in high-burden settings.

Training programmes should be incorporated into ongoing professional development for nurses and made accessible to those in rural and underserved areas, where opportunities for continuous professional growth may be scarce. Furthermore, incorporating HIV-PrEP education into nursing curricula at both undergraduate and postgraduate levels will prepare future nurses to implement HIV prevention strategies from the outset of their careers.

A barrier to PrEP implementation identified in this study is the lack of clear clinical guidelines for nurses. It is recommended that health authorities develop standardised protocols for PrEP initiation and monitoring that are straightforward and adaptable across different healthcare settings. These protocols should offer step-by-step guidance on assessing PrEP suitability for patients, managing follow-up appointments and addressing potential side effects or complications. Clear guidelines will help nurses feel more confident and reduce uncertainty when prescribing PrEP.

The study also found that some nurses hesitate to prescribe HIV-PrEP because of concerns about encouraging risky sexual behaviour. To address these concerns, training programmes should include discussions on the ethical aspects of HIV-PrEP use, supported by evidence-based facts, along with strategies for counselling patients about risky sexual conduct. Nurses should be encouraged to adopt a comprehensive approach to HIV prevention that combines PrEP with behavioural interventions, empowering patients to make informed choices about their sexual health.

The low rate of HIV-PrEP initiation among nurses in this study indicates that practical barriers, such as limited time and resources, hinder patient uptake in primary care. To address this, healthcare facilities should be provided with the necessary resources to support HIV-PrEP implementation, including additional staffing, access to PrEP medications and dedicated time for patient counselling and follow-up. Health authorities should also consider developing outreach programmes that deliver HIV-PrEP services to underserved communities, ensuring all individuals at risk of HIV can access this preventive measure.

While primary care nurses demonstrated a high level of awareness and positive attitudes towards PrEP, there are practice and knowledge gaps. It highlights the vital role played by frontline primary care providers in resource-poor settings in the prevention of HIV infection.

### Limitations

The study acknowledges the following limitations: Firstly, the study’s cross-sectional design limits the ability to establish causal relationships. Secondly, the data were collected from a single rural sub-district, meaning the views of primary care nurses cannot be generalised. Lastly, the study’s findings are based on individuals’ expressed opinions, which may result in over- or under-representation across gender groups. Future studies should monitor PrEP uptake following the intervention and explore patient perspectives on rural accessibility.

## Conclusion

This study highlights primary care nurses’ knowledge, attitudes and practices regarding HIV PrEP in rural healthcare settings. While awareness is high, knowledge gaps remain in understanding indications, contraindications and formulations of HIV PrEP. Attitudinal issues, such as misconceptions about risk compensation and resistance, emphasise the need for targeted education. Barriers, including inadequate training, unclear guidelines and time constraints, continue to limit implementation. The study’s findings recommend HIV PrEP training, the availability of standardised protocols and additional human and healthcare resources in PHC settings. Community awareness could help reduce stigma associated with PrEP use among high-risk groups.
